# Twisting of Inflatable Penile Prosthesis Tubing Leading to Device Malfunction and Required Explantation: A Rare Complication

**DOI:** 10.1155/2024/4446878

**Published:** 2024-10-15

**Authors:** Jordan Sarver, Eriel Emmer, Alex Benben, Matthew Skalak, Daniel Talley, Mazen Abdelhady

**Affiliations:** ^1^Urology Residency, Detroit Medical Center, Harper Professional Building 4160 John R St. Suite 1017, Detroit, Michigan 48201, USA; ^2^Michigan State College of Osteopathic Medicine, 965 Wilson Rd, East Lansing, Michigan 48824, USA

**Keywords:** erectile dysfunction, mechanical failure, penile prosthesis, urology

## Abstract

Erectile dysfunction (ED), the impairment of achieving and maintaining an erection for satisfactory sexual intercourse, is a common pathology that men experience for a variety of different factors. Conservative treatment for ED includes changing medications, lifestyle modifications, and psychotherapy. Pharmaceutical and nonsurgical interventions include phosphodiesterase-5 inhibitors(PDE-5i), intracavernosal medication injections, and vacuum devices. Surgical treatment options for ED have evolved over time and currently include the use of inflatable penile prosthesis (IPP) and malleable penile prosthesis. IPP insertion is usually met with good patient satisfaction. However, complications of device insertion can include corporal perforation, urethral injury, cylinder erosion or extrusion, infection, and mechanical failure, to name a few. Our patient presented with device malfunction and intraoperative assessment showed the IPP tubing twisted at the levels of the reservoir on the first operation and the level of the cylinder and scrotal pump on the second operation. The twisting of the tubing resulted in a nonfunctioning IPP as the fluid was unable to fill the cylinders resulting in an erection. The patient was managed with complete device explanation and reinsertion of a new three-piece IPP per the patient and partner's request. This is the first case report highlighting this specific complication, and we hope to provide clinicians with the resources to recognize this rare complication.

## 1. Introduction

Erectile dysfunction (ED), the impairment of achieving and maintaining an erection for satisfactory sexual intercourse, is a common pathology that men experience for a variety of different factors. These factors may include psychologic, neurologic, hormonal, vascular, or medication-induced causes [1]. Some common diseases associated with ED include diabetes, hypertension, obesity, and cardiovascular disease [1]. In addition, some lifestyle factors that contribute to ED include obesity, diabetes, and cigarette smoking [1]. When determining the cause of ED it is important to obtain a thorough history, physical exam, laboratory testing, and the use of tools such as the Sexual Health Inventory for Men (SHIM) [2]. With information from the patient's workup, the clinician can better locate the etiology of ED and appropriately guide treatment.

Conservative treatments for ED include changing chronic medications, addressing lifestyle factors, and psychotherapy [2]. Pharmaceutical and nonsurgical interventions include phosphodiesterase-5 inhibitors (PDE-5i), intracavernosal medication injections, and vacuum devices [2]. While these options are suitable for some, many patients will not respond to conservative or pharmacologic treatments. For such patients, the use of penile prosthesis surgery can be offered [2].

**Figure 1:**
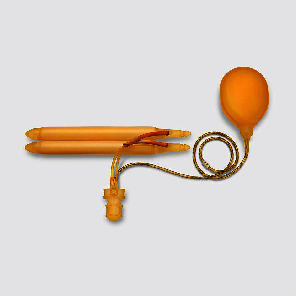
Three-piece inflatable penile prosthesis, including corporal cylinders, reservoir, and scrotal pump (AMS 700 Boston Scientific).

The use of penile implant surgery has evolved over time and currently includes the use of inflatable penile prosthesis (IPP) and malleable penile prosthesis [3]. A common device is the three-piece IPP, consisting of two intracorporal cylinders, a fluid reservoir, and a pump [3] (Figure 1) [4]. Two companies manufacture these devices and are approved in the United States: Coloplast and AMS/Boston Scientific [3]. While these devices have improved overtime, they still have associated complications. Known complications include device malfunction, cylinder erosion, cylinder crossover, device infection, urethral injury, glans ischemia, and reservoir-related complications, although others have been reported in the literature [3].

A potential complication that is not well reported in the literature is the twisting of the IPP tubing leading to device malfunction. This case report looks at a 43-year-old male who, after experiencing two malfunctioning IPPs requiring explant and reimplantation, was found to have additional IPP malfunctions due to twisting of the tubing on itself, resulting in a nonfunctioning prosthesis.

## 2. Case Report

This case is a 43-year-old male with a past medical history of a neurogenic bladder, urethral strictures, and chronic urinary retention who presented to the clinic for concerns of a malfunctioning IPP. His three-piece IPP was originally placed in September of 2012 (Coloplast Titan penile prosthesis), and he underwent two subsequent explant and reimplants for right to left midshaft crossover of the penile corpora cylinders and again for notable broken tubing between the left corporal cylinder and the pump. This first occurred 2 years later in November of 2014 with an explant and reinsertion of a three-piece IPP and subsequently after 9 years in September of 2023 with removal and reinsertion of the device.

Each time he had a three-piece IPP replaced and followed up outpatient (Coloplast Titan Touch penile prosthesis). Two months after his most recent IPP replacement (September of 2023), in November of 2023, after surgery, he then presented to the clinic. He was using his IPP postoperatively once cleared by the urologist; however, he noticed that for the past month, his IPP would not inflate properly. On examination, the IPP was difficult to inflate. He was then scheduled for IPP revision after being presented with options for observation, conversion to a malleable prosthesis, or revision and explant of his three-piece IPP.

**Figure 2:**
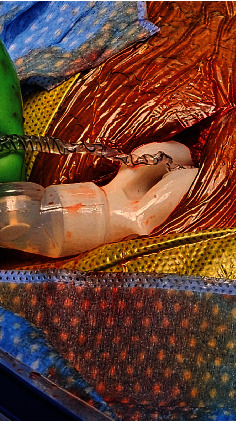
Intraoperative picturing of the twisted IPP tubing around the corporal cylinders.

**Figure 3:**
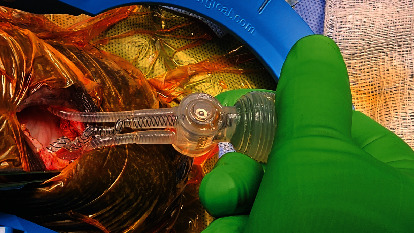
Intraoperative picturing of the twisted IPP tubing around the insertion into the scrotal pump.

Intraoperatively, the IPP was found to have a severely twisted and occluded reservoir where the tubing enters the touch pump. Upon exposing the pump, there was a significant twisting of the tubing coming from the reservoir. Shod hemostats were applied proximally and distally to the tubing connector, and the tubing connector was then disconnected. Twisted section of the tubing was untwisted and was found to have 7−10 twists in total. The surgeons reconnected the untwisted and unkinked tubing back to the pump and this functioned well. We discussed intraoperatively with the patient's partner whether to untwist and secure the tubing in the correct position or to replace a new three-piece IPP. She opted for a new replacement of a three-piece IPP (Coloplast Titan prosthesis). All portions of the device were explantated and a new three-piece IPP was placed. The patient then presented post-op 2 months after replacement of the device in January 2024 and noticed crowding of the tubing on the right at the base of the penis. He denied cycling the pump or engaging in sexual intercourse earlier than recommended. On examination, the penile cylinders were partially inflated, and the tubing was again twisted based on palpation. He was then again scheduled in January 2024 for IPP revision after being presented with options for observation, conversion to a malleable prosthesis, or revision and explant of his three-piece IPP. After the incision at the penoscrotal junction, the existing IPP was seen with severely twisted tubing which also occluded the reservoir tubing. The involved tubing was around the level of the corporal cylinders (Figure 2) and the scrotal pump (Figure 3). A second incision in the right lower abdominal quadrant was made with the delivery of the reservoir through the incision. The tubing was so severely twisted that even after untwisting it, fluid would not flow through the tubing and the pump would not function. Existing corporal cylinders were uninvolved and remained in place. The affected portions of the IPP implant were removed and replaced (Coloplast Titan Touch prosthesis). Currently, the patient is using his IPP with success and is content with the results.

## 3. Discussion

This case report highlights a rare complication of IPP insertion. Our patient presented with device malfunction and intraoperative assessment showed the IPP tubing twisted at the levels of the reservoir on the first operation and the level of the cylinder and scrotal pump on the second operation. The twisting of the tubing resulted in a nonfunctioning IPP as the fluid was unable to fill the cylinders resulting in an erection. The patient was managed with complete device explanation and reinsertion of a new three-piece IPP per the patient and partner's request. This is the first case report highlighting this specific complication, and we hope to provide clinicians with the resources to recognize this rare complication.

Some complications that exist from an IPP placement include corporal crossover, corporal perforation, urethral injury, cylinder erosion or extrusion, glans necrosis, bladder injury, visceral injury, vascular injury, hematoma formation, infection, mechanical failure, floppy glans, and reduced penile length and sensation [5]. Specifically, device malfunction may be a postoperative complication experienced by some patients [5]. Studies quote a median device survival of over 50% at a follow-up of 20 years after device placement [6]. Additionally, reported prosthesis survival rates at 5 and 10 years can reach 90.8% and 85%, respectively [5]. A malfunctioned device from mechanical failure usually requires revision surgery including complete exchange of the device [5]. Surgeons must decide when treating a device malfunction whether to replace the malfunctioned component versus the entire device as a whole. When considering the individual pieces of the malfunctioned prosthesis (i.e., cylinders, reservoir, and tubing), there have been several reported reservoir complications in the literature. This includes damage to surrounding tissue during placement, reservoir autoinflation, and herniation of the reservoir [6]. However, isolated complications of the IPP tubing, as in this reported case, are rare. One reported complication is tubing erosion as reported in a case where 14 years after device insertion, the patient reported a portion of the tube connecting the pump to the right cylinder eroding through the skin [7]. The topic of IPP-tubing complications leading to mechanical failure needs to be better studied and reported in the literature.

One prior case report exists in the literature regarding similar, albeit not identical, device malfunction. The surgeons in this case had a patient with device malfunction due to an abnormally rotated cylinder. The left cylinder was abnormally rotated 180 degrees, with this defect likely occurring during the manufacturing process [8]. This was managed by operative intervention to cut and reconnect the tubing between the cylinders and pump with the placement of a new cylinder and pump [8]. The advent of kink–resistant IPP tubing around 1980–1990 has allowed for better prosthetic durability [9]. Although these advances to improve product structures exist, patients, such as the one in this study, may still require prosthetic revision after complications arise [9, 10]. These complications may include fracture or twisting of the tubing leading to device malfunction [10].

Our case presented here was managed by device explant and reinsertion due to the nonfunctioning device and the patient's wishes to continue to have erections good enough for sexual intercourse. Other options for management in a patient who presents with this complaint of mechanical failure could be explant and conversation to malleable prosthesis, observation if the device does not display signs of infection and erosion, removal of the device if not desired by the patient, or revision of the implant and tubing without complete explant of the device. Postoperative instructions and expectation management between the patient and surgeon are important.

The fact that the patient in this case had his IPP tubing twist multiple times leading to malfunction is concerning. Interestingly, in this case, the patient denied cycling his pump too early before being cleared by his surgeon for sexual intercourse. However, we still believe there was some information the patient may have left out. There is the possibility the patient was using the implant too early or being involved with too aggressive sexual intercourse. This may have resulted in damage or excessive force being placed on the IPP tubing leading to twisting and malposition within the patient. We believe this could be the reason this patient or someone alike would have this issue with the device. The tubing is designed to prevent kinks or twisting, and despite multiple surgical revisions, the patient still had this rare complication. Continued discussion between all patients and their surgeons is important in considering patient satisfaction and surgical outcomes surrounding penile prostheses. Knowledge that patient's may not follow postoperative directions correctly or may have specific sexual preferences may help open discussion on why rare complications may be seen that are not obviously due to surgical technique, but instead postoperative use.

## 4. Conclusion

This case report highlights a rare complication of IPP insertion. Our patient presented with device malfunction, and intraoperative assessment showed the IPP tubing twisted at the levels of the reservoir on the first operation and the level of the cylinder and scrotal pump on the second operation, leading to a nonfunctioning prosthesis. This issue was managed by device explant and reinsertion. IPP insertion is usually met with good patient satisfaction; however, we hope to provide clinicians with the resources to recognize and manage this rare complication.

## Data Availability

Data sharing is not applicable to this article as no new data were created or analyzed in this study.
